# Supplementary figures and images for: A Lipid Nanoparticle-Formulated Self-Amplifying RNA Rift Valley Fever Vaccine Induces a Robust Humoral Immune Response in Mice
Source: Vaccines (Basel). 2024 Sep 24;12(10):1088. doi: 10.3390/vaccines12101088 (PMC11511412; doi:10.3390/vaccines12101088)

Figure 3A

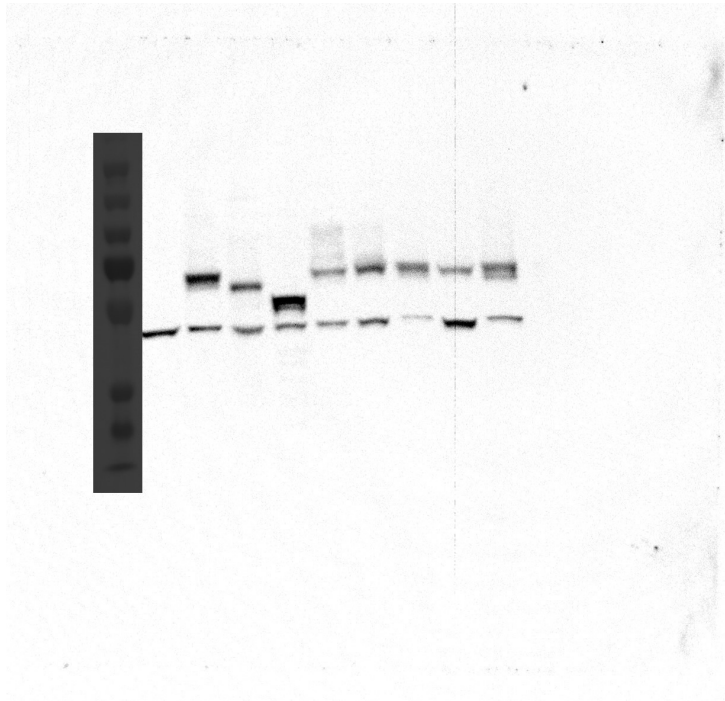

Figure 3B

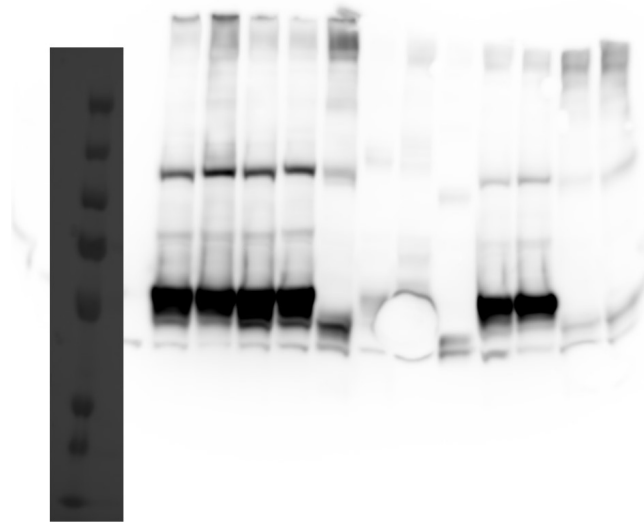

Figure 4B

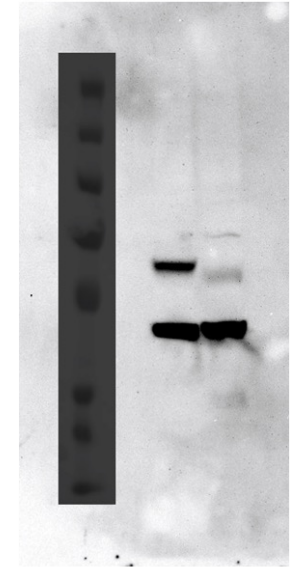

Figure 3C

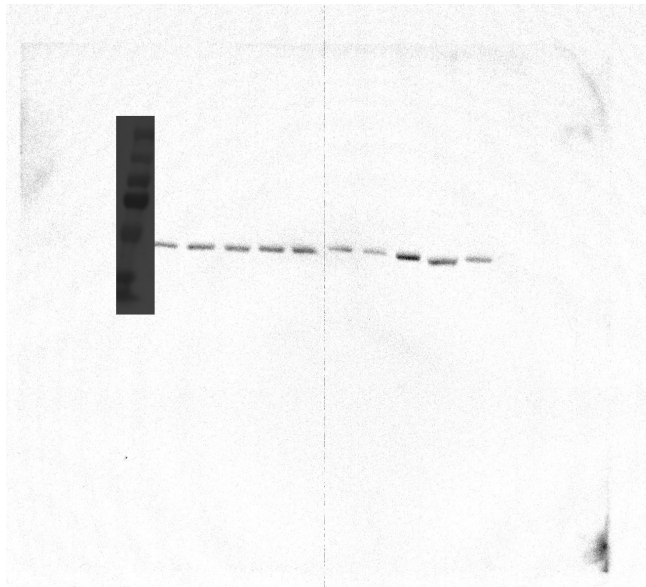

Figure 3D

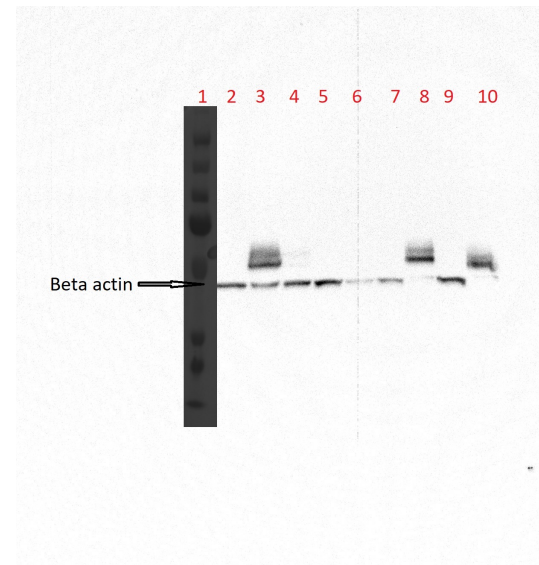

Supplement: Supplementary file 1 [file vaccines-12-01088-s001.zip › Figure S2 SDS-PAGE Western blot raw data images.pdf]
